# Prediction of Srebp-1 as a Key Target of Qing Gan San Against MAFLD in Rats via RNA-Sequencing Profile Analysis

**DOI:** 10.3389/fphar.2021.680081

**Published:** 2021-07-05

**Authors:** Bendong Yang, Jingyue Sun, Shufei Liang, Peixuan Wu, Rui Lv, Yanping He, Deqi Li, Wenlong Sun, Xinhua Song

**Affiliations:** ^1^ School of Life Sciences, Shandong University of Technology, Zibo, China; ^2^ Key Laboratory of Novel Food Resources Processing, Ministry of Agriculture and Rural Affairs/Key Laboratory of Agro-Products Processing Technology of Shandong Province/Institute of Agro-Food Science and Technology, Shandong Academy of Agricultural Sciences, Jinan, China

**Keywords:** traditional Chinese medicines, SREBP-1, Qing Gan San, RNA sequencing, metabolism-associated fatty liver disease

## Abstract

Metabolism-associated fatty liver disease (MAFLD) is the most common chronic liver disease worldwide, and the use of traditional Chinese medicines (TCMs) to treat this disease has attracted increasing attention. The Qing Gan San (QGS) formula comprises *Polygonatum sibiricum,* the peel of *Citrus reticulata Blanco,* the leaves of *Morus alba L, Cichorium intybus, Glycyrrhiza uralensis Fisch,* and *Cirsium setosum.* The present study aimed to uncover the anti-hyperlipidaemic effects, hepatic fat accumulation-lowering effects and mechanisms of QGS in high-fat diet-induced MAFLD rats. QGS significantly reduced the levels of total cholesterol and triglycerides in both serum and liver tissue and partially protected hepatic function. Additionally, QGS significantly ameliorated hepatic lipid accumulation with histopathology observation, as demonstrated by H&E and oil red O staining. RNA sequencing was used to further investigate the key genes involved in the development and treatment of MAFLD. Hierarchical clustering analysis showed that the gene expression profiles in rats with MAFLD were reversed to normal after QGS treatment. QGS had 222 potential therapeutic targets associated with MAFLD. Enrichment analysis among these targets revealed that QGS affected biological functions/pathways such as the regulation of lipid metabolic processes (GO: 0019216) and the non-alcoholic fatty liver disease pathway (hsa04932), and identified Srebp-1 as a key regulator in the synthesis of cholesterol and triglycerides. Subsequently, both immunofluorescence and Western blot analyses demonstrated that QGS suppressed the transfer of Srebp-1 to the nucleus from the cytoplasm, suggesting that the activation of Srebp-1 was inhibited. Our study reveals the effects and mechanisms of QGS in the treatment of MAFLD and provides insights and prospects to further explore the pathogenesis of MAFLD and TCM therapies.

## Introduction

Metabolism-associated fatty liver disease (MAFLD), characterized by hepatic lipid accumulation without excessive alcohol consumption, has become a common metabolic liver disease with a global prevalence of ∼25% (Friedman et al., 2018). Recent studies have demonstrated that metabolic syndrome, which typically involves obesity, hyperglycaemia, dyslipidaemia, and hypertension, is a critical risk factor for the progression of MAFLD (Huang, 2009). Additionally, MAFLD is strongly associated with chronic diseases such as cardiovascular disease (CVD) and type 2 diabetes mellitus (T2DM) (Mikolasevic et al., 2016). Although knowledge concerning the pathogenesis of MAFLD has expanded rapidly in recent years, approved drugs by the Food and Drug Administration are lacking. Exercise and diet remain dominant options for treating MAFLD, and few people can maintain a regulated lifestyle for a long time (Friedman et al., 2018). Thus, more available therapeutic strategies are required to suit the various demands of MAFLD patients.

Traditional Chinese medicines (TCMs) are widely used to prevent and treat chronic diseases in China. The QGS formula is effective for MAFLD treatment through long-term clinical experience. The QGS formula comprises six herbs: *Polygonatum sibiricum*, the peel of *Citrus reticulata Blanco*, the leaves of *Morus alba L*, *Cichorium intybus*, *Glycyrrhiza uralensis Fisch*, and *Cirsium setosum*. These herbs have good effects against hepatic steatosis and related pathologies, but few studies have investigated the polysaccharide constituents of herbs that influence mechanisms against MAFLD (Yang et al., 2015; Guo et al., 2016; Peng et al., 2018; Wu et al., 2018; Wang et al., 2019; Gou et al., 2020). In our study, the efficacy of QGS polysaccharide in treating MAFLD was comprehensively estimated.

In recent decades, RNA sequencing techniques involving high-throughput screening and the identification of targets have greatly expanded the application of TCMs (Arendt et al., 2015). In a previous study, Huang-Qi San, a Chinese herbal formula, improved lipid accumulation and provided protective effects against hepatic steatosis in high-fat diet-fed rats via the analysis of RNA sequence data (Li et al., 2020). Additionally, the GeneCards database can enable a deep understanding of how TCMs influence important targets involved in the occurrence and progression of MAFLD (Fishilevich et al., 2017). Herein, RNA sequencing was used as an efficient strategy to identify target genes and metabolism.

The present study was designed to clarify the efficacy of QGS against MAFLD in rats. The compositions and structures of QGS were analyzed by ion chromatography and infrared radiation, respectively. Using a rat model of MAFLD induced by high-fat diet feeding, we investigated the effects of QGS in rats with MAFLD and clarified the potential underlying mechanisms via the combination with RNA sequencing and the GeneCards database.

## Materials and Methods

### Preparation and Quality Control of QGS

For QGS preparation, *Polygonatum sibiricum*, the leaves of *Morus alba L, Cichorium intybus*, *Cirsium setosum,* the peel of *Citrus reticulata Blanco*, and *Glycyrrhiza uralensis Fisch* were purchased from Tongrentang Chinese Medicine Co., Ltd. (Beijing, China) and then ground in a grinder and mixed at a 15:10:10:10:6:6 ratio. The processing steps were performed as described in a previous study with minor modifications (Zhang et al., 2018). Briefly, the powder mixture was extracted with boiling distilled water three times (1:10, w/v), and then the supernatant was deoiled with ligroin (10:1, v/v) to remove small molecules. Subsequently, the solution was concentrated and precipitated with 75% ethanol. After being stored for 24 h, the mixture was centrifuged at 4°C at 4,500 rpm/min for 10 min, and the extract was dried with a vacuum freeze dryer for further analysis. The production was calculated to be 36.81%, and measurement of the main components (polysaccharides, polyphenols, proteins and galacturonic acid) was performed.

Quality control was also conducted by ion chromatography using a Dionex CarboPac^TM^ PA20 (3 × 150) column and an electrochemical detector. The mobile phases comprised 1) H_2_O and 2) 250 mM NaOHC (50 mM NaOH and 500 mM NaOAc). The flow rate was 0.3 ml/min, and the column temperature was set to 30°C. Glucose, galactose, chlorinated galactosamine, rhamnose, arabinose, and xylose were prepared as standards to establish calibration curves to perform quantitative analysis. To prepare the QGS solution, 0.01 g of QGS was weighed and dissolved in a volume of 10 ml of trifluoroacetic acid for 3 h at 120°C. Subsequently, the aqueous acid solution was dried under nitrogen. The powder was redissolved in 5 ml of double-distilled water, and then 100 µL was pipetted into 900 µL of deionized water. The mixture was centrifuged at 12,000 rpm for 5 min, and the supernatant was used for ion chromatography analysis. Additionally, infrared radiation spectra were detected using a Fourier transform infrared apparatus, the wavenumbers of which are reported in cm^−1^. The polysaccharide molecular weight was investigated using high-performance gel permeation chromatography.

### Animal Experiment and Sample Collection

Male Sprague-Dawley rats (8 weeks old, weighing 180–220 g) were purchased from the Shandong Laboratory Animal Center (Jinan, China) with the permission number SCXK 2014-0007. All the animal procedures were performed in accordance with the Guidelines for the Care and Use of Laboratory Animals of Shandong University of Technology and were permitted by the Animal Ethics Committee of Shandong University of Technology. The rats were first housed at 25 ± 0.5°C under a 12 h light/dark cycle for adaptation for one week. Next, the rats were randomly divided into three groups (*n* = 6 per group): normal control (NC) group, high-fat diet (HFD) group, and QGS treatment (QGS) group. The high-fat diet comprised 20% protein, 35% carbohydrate, and 45% fat as a percent of total calories (Supplementary Table S1). The rats in the HFD and QGS groups were given a high-fat diet for 12 weeks continuously, while the NC group was fed a normal diet (Keaoxieli, Beijing, China). The rats in the QGS group received 100 mg/ml of QGS extract at 8:00 am for the last 8 weeks until sacrifice, while the rats in the NC and HFD groups received equal amounts of saline as a control.

During the last week, the body weight and food intake of the rats in each group were recorded. At the end of the experimental period, blood samples were collected and centrifuged at 3,000 rpm for 5 min at 4°C. The samples were then preserved at −80°C for further analysis. Liver samples were also collected, frozen with liquid nitrogen and stored at −80°C.

### Measurement of Serum Biochemical Indexes

After fasting for 16 h, the TC and TG levels in both plasma and liver samples, as well as the low-density lipoprotein cholesterol (LDL-C) and high-density lipoprotein cholesterol (HDL-C) levels in plasma, were assayed immediately using commercial kits (Jiancheng, Nanjing, China). Additionally, the plasma alanine aminotransferase (ALT) and aspartate aminotransferase (AST) levels were also determined directly using commercial kits.

### Histopathological Analysis

Hematoxylin and eosin (H&E) staining was performed to observe the degree of fat accumulation in the liver. The liver tissues were fixed with formalin, embedded in paraffin, cut into 3 µm-thick slices, stained with H&E and then observed using a light microscope at ×40.0 magnification. Additionally, liver cryosections were stained with oil red O and counterstained with hematoxylin to visualize the lipid droplets using an optical microscope at ×40.0 magnification.

### RNA Sequencing Analysis

RNA sequencing was conducted using homogenized liver tissue (three replicates each for the NC, HFD, and QGS groups). Total RNA was extracted using TRIzol reagent (Qiagen, Germany) according to the manufacturer's instructions. RNA sequence library construction was performed using the Illumina TruSeq^TM^ RNA Sample Prep Kit method. The concentration, purity, and quality of the RNA were evaluated using the Agilent Bioanalyzer system (Agilent, United States) with the criteria of a 28S:16S ratio >1.5 and a 260/280 absorbance ratio between 1.8 and 2.1. Subsequently, the Illumina NovaSeq 6,000 platform (LC Science, United States) was employed for quantification and sequencing according to a standard sequencing protocol.

The sequencing data were quality controlled using fastx_toolkit_0.0.14 to generate clean data. The clean data were mapped to the rat reference genome (*Rattus*_*norvegicus*, version Rnor_6.0) using HISAT2 and then were assembled via StringTie software. Next, transcript quantification was performed using RSEM with the FPKM method to produce read counts. The read counts were determined to generate a gene expression profile with DESeq2, in which the default filter conditions were used (p-adjust < 0.05 and | log2 FC | ≥ 1).

### Protein Isolation and Western Blot Analysis

As previously described (Sun et al., 2020), sterol regulatory element-binding protein-1 (Srebp-1) and Lamin B1 or β-actin protein in both the nucleus and cytoplasm were extracted with kits, and the protein samples were separated by 10% SDS–polyacrylamide gel electrophoresis. Next, the protein samples were transferred to a PVDF membrane (0.22 µm), which was blocked with 5% skim milk for 4 h at room temperature. Subsequently, the membrane was incubated with primary antibodies, including Srebp-1 (1:1,000), β-actin (1:2,000), and Lamin B1 (1:2,000), which were diluted in Tris-buffered saline with Tween-20 containing 5% skim milk at 4°C overnight. The immune complexes were recognized by a secondary antibody (1:2,000) conjugated to horseradish peroxidase (ProteinTech, Chicago, United States). Peroxidase activity was visualized using an enhanced chemiluminescence kit (Solarbio, Beijing, China). Densitometric analysis of the immunoblots was performed using ImageJ software.

### Immunofluorescence Staining

Liver sections were fixed with 4% paraformaldehyde for 15 min at 37°C. Next, the sections were blocked with 2% BSA for 30 min. Subsequently, the sections were incubated with an anti-Srebp-1 antibody (1:100) overnight at 4°C. The sections were then washed and re-incubated with an FITC-labelled secondary antibody (1:100) (ProteinTech, Chicago, United States) for 1 h. Finally, immunofluorescence was observed using a fluorescence microscope after staining with DAPI for 5 min.

### Statistical Analysis

Statistical analysis was conducted using GraphPad Prism 8, and the results were presented as means ± standard error of the mean. One-way ANOVA was used for multiple comparisons. Post-hoc testing was conducted using Dunnett’s test method. Significant differences were accepted for *p < 0.05, **p < 0.01, and ***p < 0.001.

## Results

### Determination and Analysis of QGS Extract Components

The determination of the main components of the QGS extract is essential to clarify the mechanisms. The QGS extract was collected, and the contents of polysaccharides, polyphenols, proteins and galacturonic acid were assessed. The ratio of QGS polysaccharides was up to 75.5%, and the ratio of galacturonic acid was 18.1% in QGS polysaccharides, while the polyphenols and proteins were only 6.5 and 12.1%, respectively (Table 1).

**TABLE 1:** Polysaccharide, polyphenol, protein, and glycuronic contents. The data are presented as means ± SEM.

Term	Content (mg/ml)	Sample concentration (mg/ml)	Ratio (%)
Polysaccharides	0.0755 ± 0.002	0.1	75.5
Polyphenols	0.0065 ± 0.001	0.1	6.5
Protein	0.0121 ± 0.001	0.1	12.1
Glucuronic acid	0.0137 ± 0.001	0.1	13.7

**FIGURE 1:**
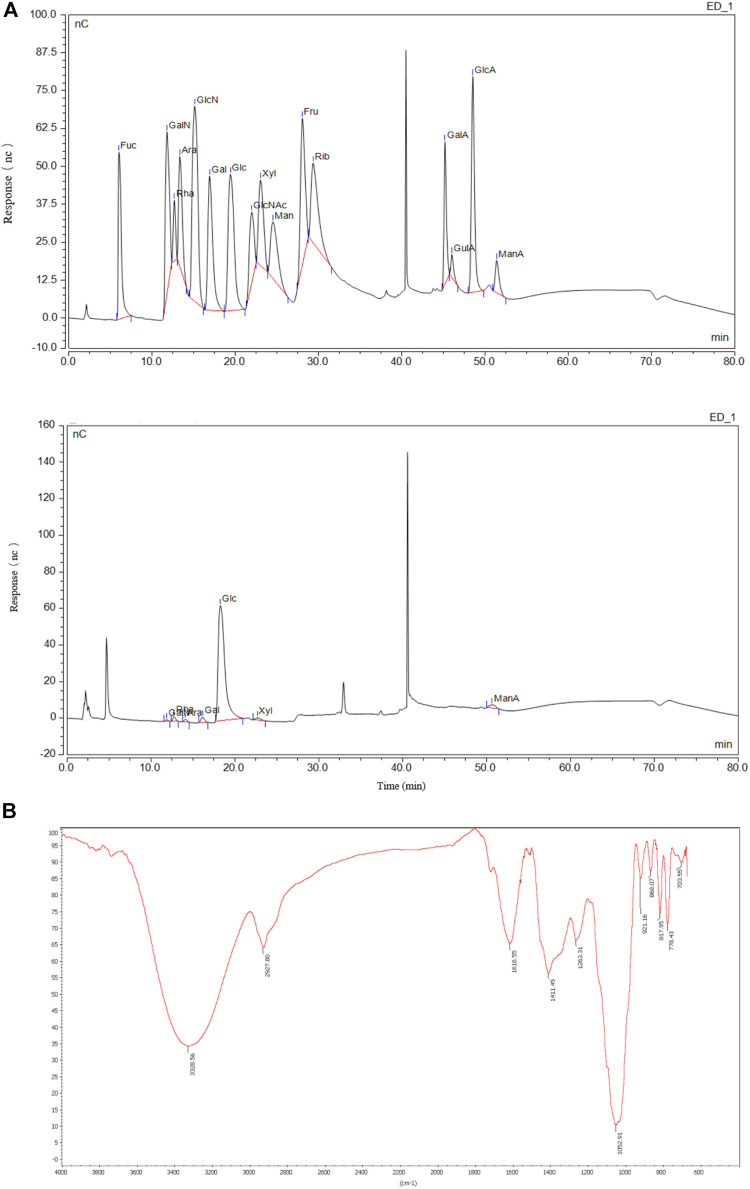
Monosaccharide compositions and structure of QGS. **(A)** The monosaccharide standard (upper panel) and QGS sample (lower panel) were measured with HPIEC. **(B)** The structure of QGS was determined by fourier transform infrared spectroscopy.

Additionally, the monosaccharide composition and polysaccharide linkage mode of QGS were investigated. The monosaccharide composition of QGS was detected using an ion spectrometer (Thermo Fisher, United States) (Figure 1A). The results suggested that the glucose: xylose: galactose: galactosamine hydrochloride: rhamnose: arabinose molar ratio was 0.896:0.028:0.024:0.018:0.014:0.014. Thus, glucose is recognized as the main monosaccharide in QGS polysaccharides. Additionally, high-performance gel permeation chromatography with a calibration curve of dextran standards (log MW = −0.2078x + 12.968; *R* ^2^ = 0.993) calculated that the average molecular weight was 5,735 kDa. Furthermore, Fourier transform infrared spectroscopy identified the bands at 868 cm^−1^ and 817.96 cm^−1^ as the β-glycosidic bond and α-glycosidic bond deformation modes, respectively.

### QGS Ameliorates Dyslipidaemia in MAFLD Model Rats

**FIGURE 2:**
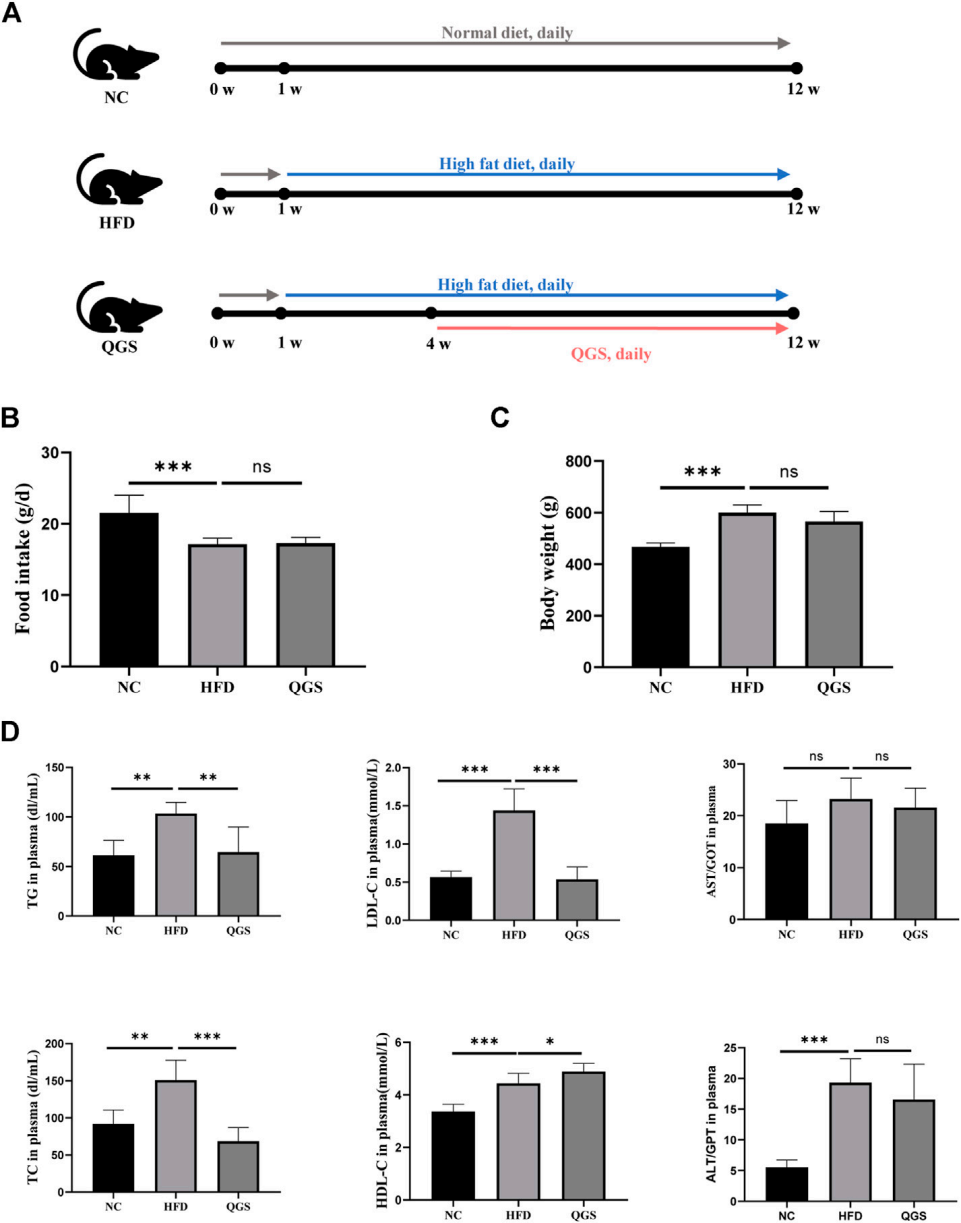
Effects of QGS on MAFLD in rats. **(A)** Flow cytometry. **(B)** Food intake of rats at the 8th week. **(C)** Body weight of rats at the 8th week. **(D)** Measurements of TC, TG, LDL-C, HDL-C, AST, and ALT in plasma. The data are presented as means ± SEM; ns, no significant difference, *p < 0.05, **p < 0.01, ***p < 0.001, vs. the HFD group.

The design and procedures of the animal experiment are shown in Figure 2A *.* Food intake was decreased in the HFD group compared with that in the NC group, while no difference was found after QGS treatment. Additionally, the body weight was significantly increased in the HFD group compared with that in the NC group, and it was slightly reduced by QGS intervention (Figures 2B,C). Because MAFLD is closely associated with dyslipidaemia, the plasma lipid levels in each group were measured. Compared with the NC group, the HFD group exhibited markedly higher plasma levels of both TC and TGs (Figure 2D). By contrast, the levels of TC and TGs were reduced significantly by QGS administration. Additionally, the LDL-C level showed trends toward growth in the HFD group that was reversed following QGS intervention. The levels of HDL-C were increased in the HFD group induced by a high-fat diet and continued to grow with QGS administration. These results suggest that QGS may attenuate dyslipidaemia induced by a high-fat diet.

### QGS Regulates Excessive Lipid Accumulation in the Livers of MAFLD Rats

**FIGURE 3:**
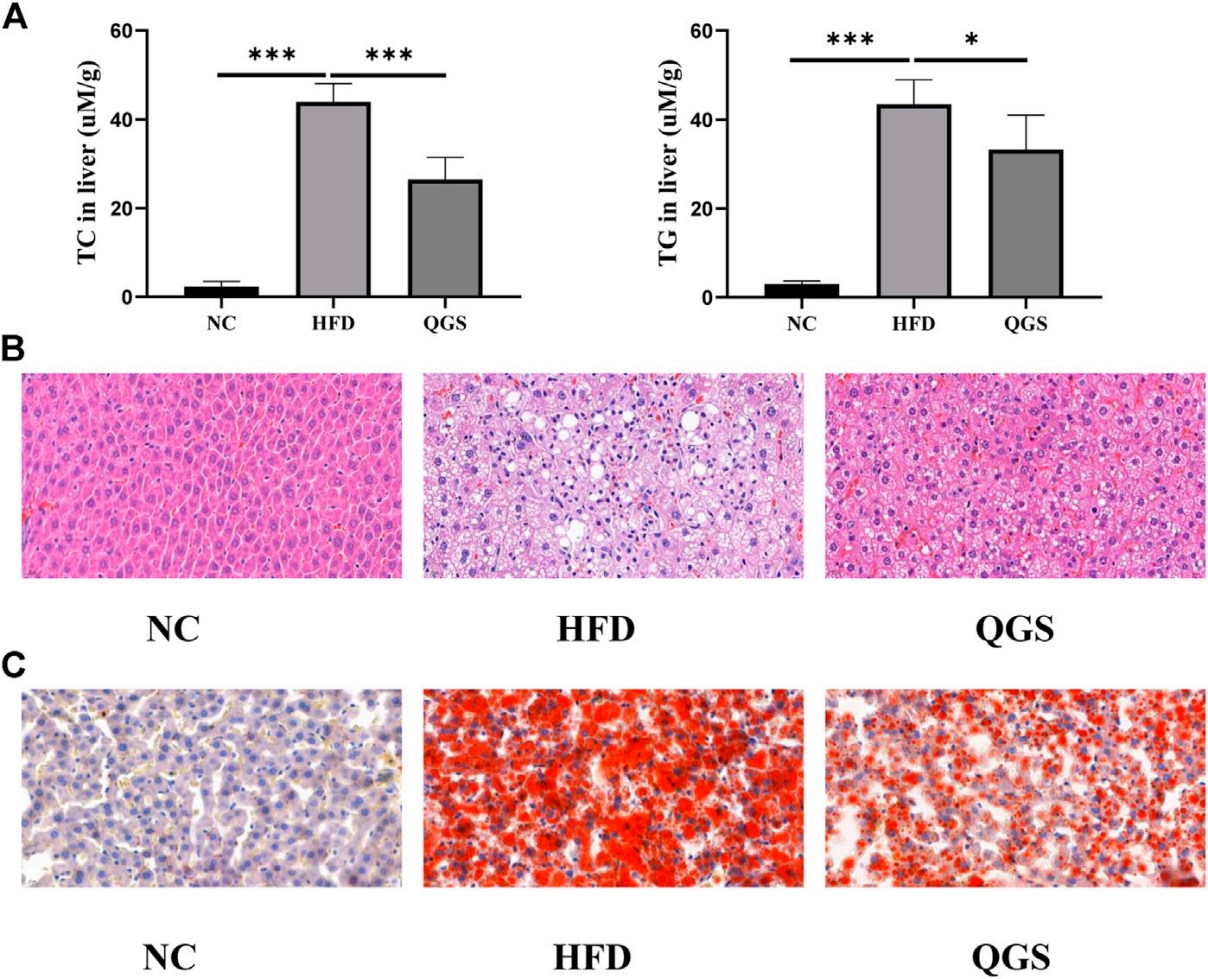
Levels of lipid accumulation in the liver. **(A)** Levels of TC and TG in the liver. **(B)** Hematoxylin and eosin staining of liver tissue at ×40.0 magnification. **(C)** Oil red O staining of liver tissue at ×40.0 magnification. The data are presented as means ± SEM. ns, no significant difference. *p < 0.05, **p < 0.01, ***p < 0.001, vs. the HFD group.

Excessive fat accumulation in the liver is a dominant sign of MAFLD. We investigated the levels of TC and TGs in the liver. QGS obviously attenuated the degrees of TC and TGs in the liver (Figure 3A). Additionally, H&E and oil red O staining were employed for histopathological observation. Histopathological change in the liver was observed between the HFD and NC groups (Figures 3B,C). More lipid droplets were observed in the HFD group than in the NC group. Daily oral administration of QGS for 8 weeks greatly ameliorated the abovementioned pathological abnormalities caused by high-fat diet feeding. Thus, we conclude that QGS could partially ameliorate hepatic lipid accumulation.

### Hepatic Gene Expression Profiles Among the NC, HFD, and QGS Groups

**FIGURE 4:**
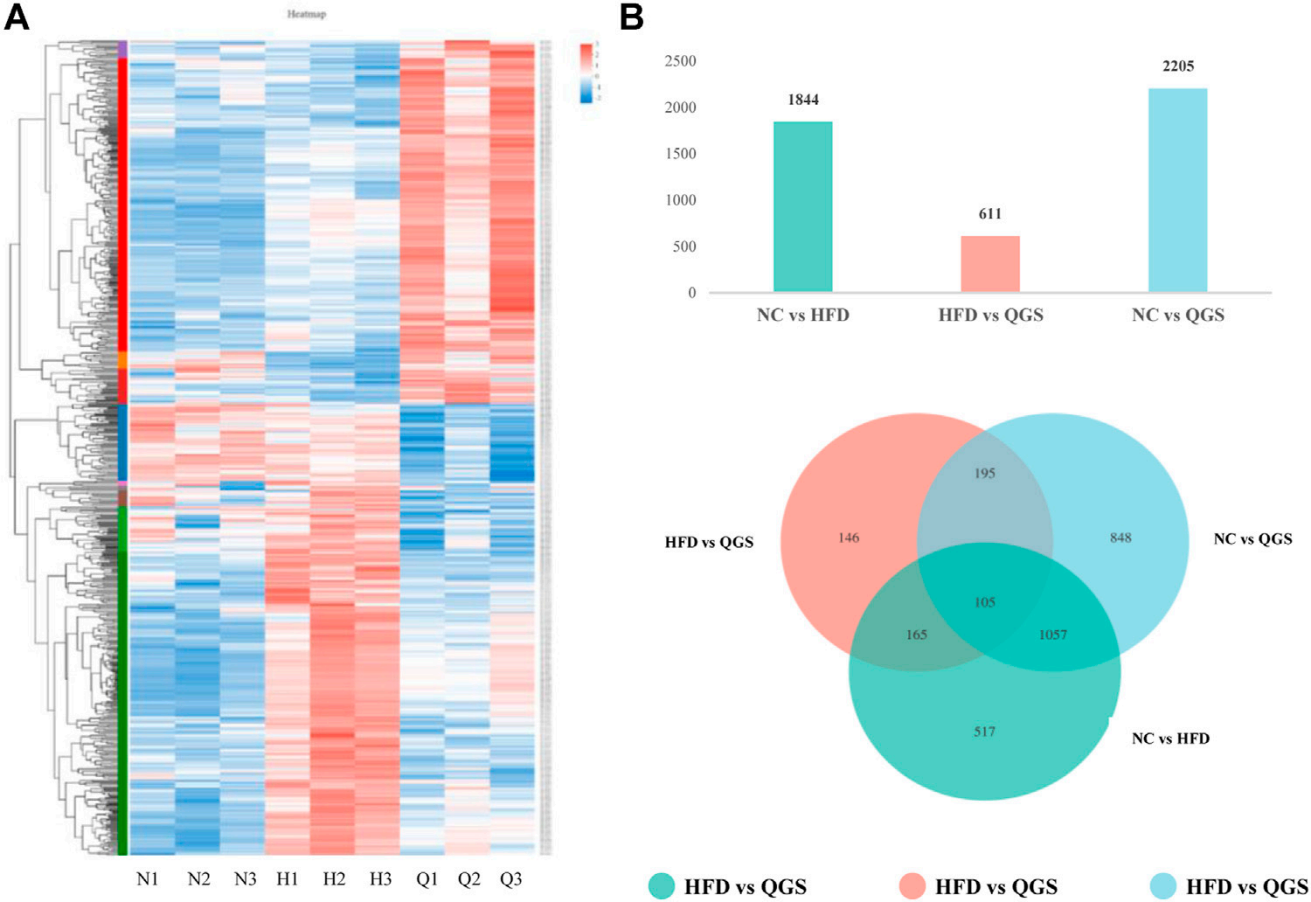
Differential expression gene analysis. **(A)** Hierarchical clustering. Red represents high expression of genes, while blue represents low expression of genes. The left part is a dendrogram of gene clustering, and the lower part is the name of the sample (N, NC group; H, HFD group; Q, QGS group). **(B)** The upper panel presents numbers in three pairwise comparisons; the lower panel shows the Venn diagram showing the number of DEGs in the three pairwise comparisons.

Raw data were loaded into NCBI (BioProject ID: PRJNA726732), and a normalized gene expression matrix was produced and submitted for further analysis (Supplementary Table S4). Gene expression profile analysis was performed using hierarchical clustering. The gene expression profile trend of the QGS group was closer to that of the NC group than to that of the HFD group (Figure 4A). According to methods described in previous reports (Hong et al., 2019), 3031 differential genes were identified using RESM software (P-adjust ＜ 0.05 and | logFC |＞ 1) in three pairwise comparisons: NC vs. HFD, HFD vs. QGS, and NC vs. QGS. The results are shown with a Venn diagram (http://www.funrich.org/) in Figure 4B. Furthermore, when our RNA sequencing data intersected with target genes involved in MAFLD from the GeneCards database (1,051 genes, searched with the key word “MAFLD”; Supplementary Table S2), 222 differentially expressed genes (DEGs) were screened and identified as potential therapeutic targets for MAFLD at subsequent analysis (Supplementary Table S3).

### GO Functional Enrichment and KEGG Pathway Analysis

**FIGURE 5:**
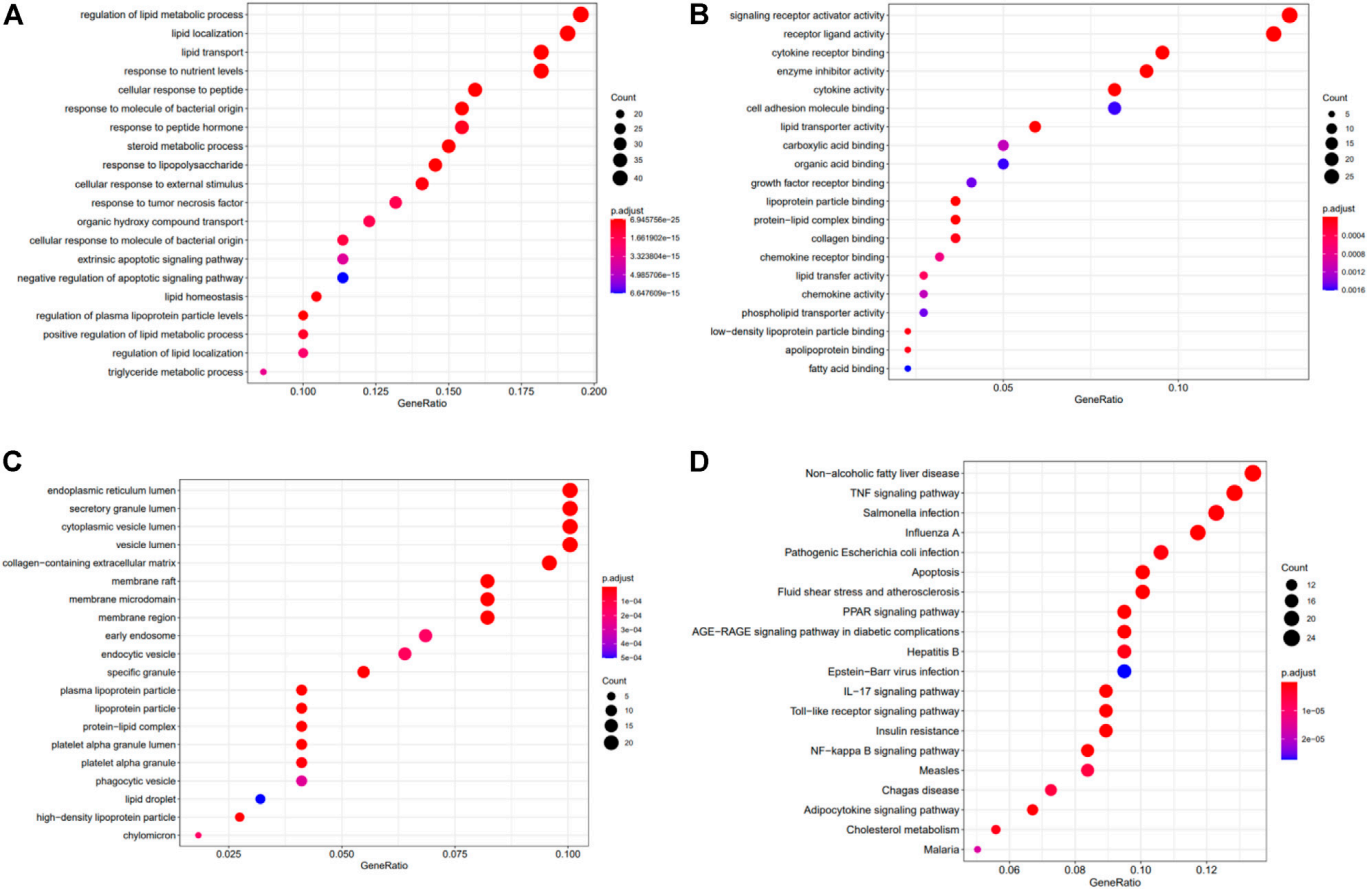
The results of GO and KEGG analyses of the crosstalk genes. GO analysis of the 222 hub genes comprised three aspects: **(A)** biological process, **(B)** cellular components, and **(C)** molecular function. **(D)** KEGG pathway analysis of the 222 potential target genes.

The 222 DEGs were subjected to Gene Ontology (GO) functional enrichment and Kyoto Encyclopedia of Genes and Genomes (KEGG) pathway enrichment analyses (Figure 5). The terms regulation of lipid metabolic process (GO: 0019216), lipid localization (GO: 0010876), and response to nutrient levels (GO: 0031667) were significantly enriched in the biological process category. The terms endoplasmic reticulum lumen (GO: 0005788), secretory granule lumen (GO: 0034774), and cytoplasmic vesicle lumen (GO: 0060205) were enriched in the cellular component category. The terms signaling receptor activator activity (GO: 0030546), receptor ligand activity (GO: 0048018), and cytokine receptor binding (GO: 0005126) were enriched in the molecular function category. KEGG pathway enrichment analysis was also performed using these DEGs and revealed that the non-alcoholic fatty liver disease pathway (hsa04932) and the tumor necrosis factor (TNF) signaling pathway (hsa04668) terms were enriched for 24 genes and 23 genes, respectively (Table 2).

**TABLE 2:** The results of GO and KEGG enrichment analyses.

Term	ID	Count
**Biology process**		
Regulation of lipid metabolic process	GO: 0019216	44
Response to nutrient levels	GO: 0031667	44
Lipid localization	GO: 0010876	41
**Molecular function**		
signaling Receptor activator activity	GO: 0030546	31
receptor ligand activity	GO: 0048018	30
cytokine Receptor binding	GO: 0005126	22
**Cellular component**		
secretory granule lumen	GO: 0034774	25
cytoplasmic vesicle lumen	GO: 0060205	25
vesicle lumen	GO: 0031983	25
**KEGG enrichment analysis**		
Non-alcoholic fatty liver disease	hsa04932	24
TNF signaling pathway	hsa04668	23
*Salmonella* infection	hsa05132	22

### Screening of Srebp-1 as a Key Regulator of Lipogenesis

**FIGURE 6:**
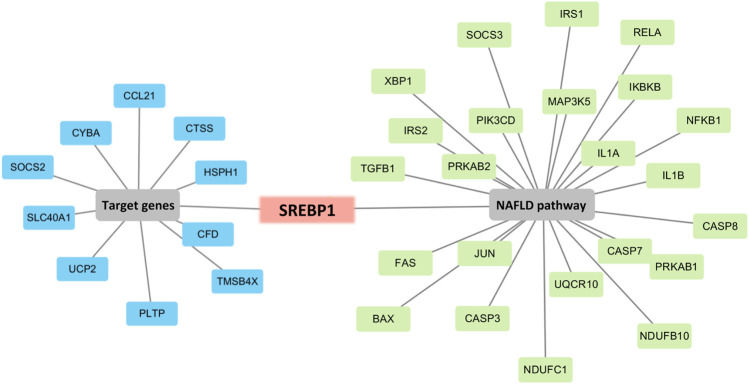
Screening of the key regulated gene. Combining target genes with MAFLD pathway genes, only Srebp-1 was screened out. The blue box represents target genes, the green box represents genes participating in the MAFLD pathway, and the red box represents genes involved in both the target genes and the MAFLD pathway.

To determine the hub genes, we examined the intersection of genes participating in the MAFLD pathway and target genes. Eleven genes were upregulated in HFD and downregulated with QGS treatment or downregulated in HFD and upregulated with QGS treatment, and were collected as target genes (Table 3). Only Srebp-1 was upregulated by high-fat diet feeding, downregulated after QGS intervention and involved in the MAFLD pathway (Figure 6). Therefore, Srebp-1 may be a key regulator associated with MAFLD treatment after QGS intervention.

**TABLE 3:** Screening of target genes.

Treatment comparison	Gene count	Gene information
NC-HFD: Upregulated and HFD-QGS: Downregulated	9	SREBP1, CYBA, PLTP, YCP2, CTSS, CFD, CCL21, TMSB4X, SLC40A1
NC-HFD: Downregulated and HFD-QGS: Upregulated	2	HSPH1, SOCS2

### Validation of the Western Blot and Immunofluorescence Analyses

**FIGURE 7:**
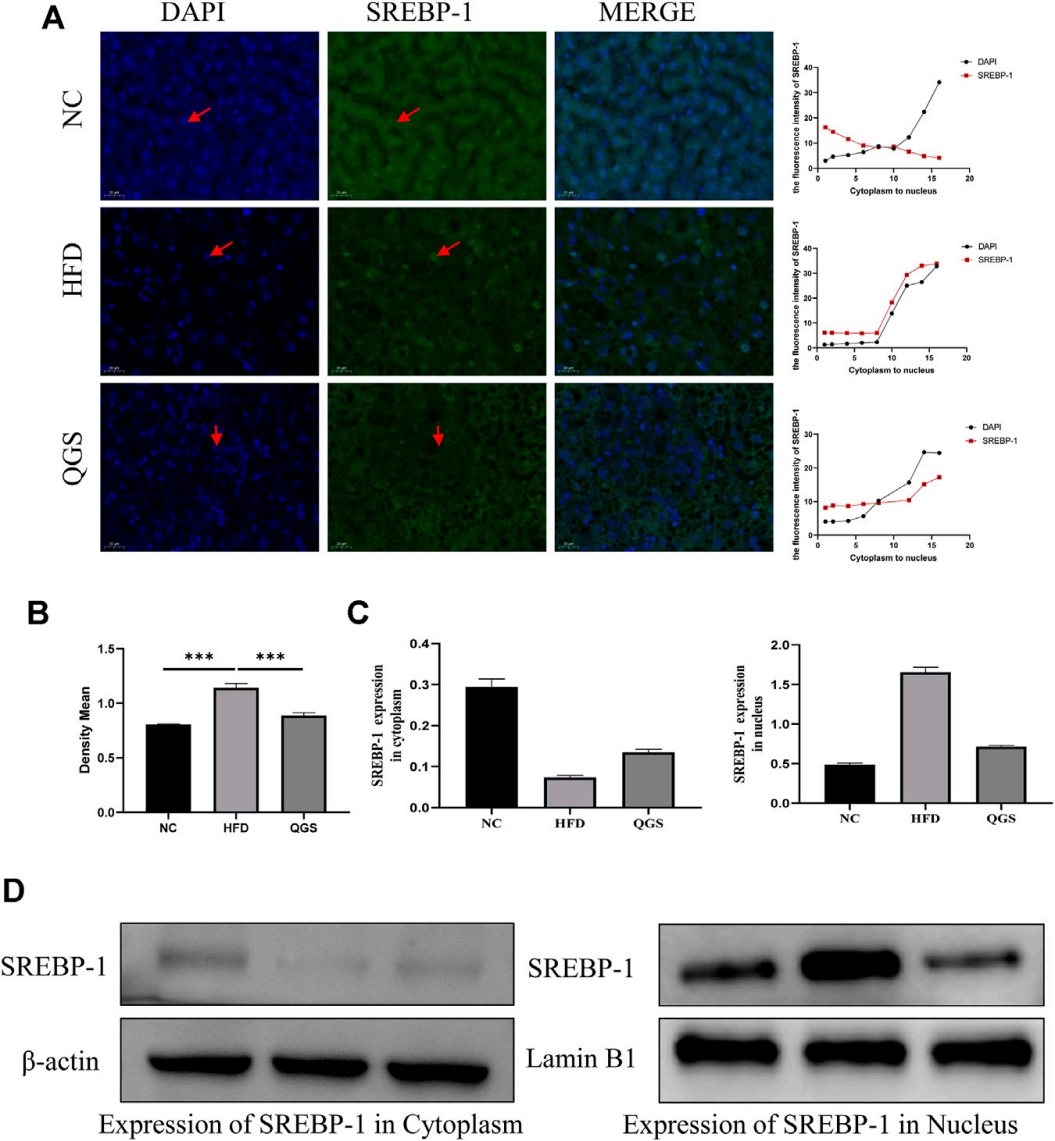
Effects of QGS on the expression of Srebp-1 in the cytoplasm and nucleus. **(A)** Immunofluorescence staining for Srebp-1 in liver tissue (original magnification, ×200). The line chart presents the trend of expression from the cytoplasm to the nucleus. **(B)** The relative protein expression in immunohistochemistry was quantified using ImageJ software. The results are represented as the density mean (density mean = integrated density/area). **(C,D)** The expression levels of Srebp-1 in the cytoplasm and nucleus were detected by Western blotting. β-actin and Lamin B1 were used as loading controls.

The expression and localization of Srebp-1 were verified by Western blotting and immunofluorescence. A clear difference was observed between each group: the expression of Srebp-1 was higher in the HFD group than in the NC group, whereas the expression of Srebp-1 was decreased after QGS treatment (Figures 7A,B). A significantly greater proportion of Srebp-1 was recruited to the nucleus in the HFD group than in the NC group, but intervention with QGS reversed this trend. The expression of Srebp-1 in the nucleus was significantly increased in the HFD group (Figures 7C,D). QGS treatment significantly decreased the expression of Srebp-1 in the nucleus. Moreover, the expression of Srebp-1 in the cytoplasm was lower in the HFD group than in the NC and QGS groups. These results indicate that QGS treatment changes the expression of Srebp-1 in the nucleus and cytoplasm.

## Discussion

MAFLD, formerly known as NAFLD, which encompasses a spectrum of diseases, including simple steatosis, non-alcoholic steatohepatitis, cirrhosis, and hepatocellular carcinoma, has become a major hepatic metabolic disease worldwide (Tilg, 2017). MAFLD is closely associated with several diseases, including chronic kidney disease, T2DM, and CVD. The pathogenic causes of MAFLD are multifactorial, and known factors include genetics, the environment, and metabolic syndrome, which contribute to the occurrence and progression of MAFLD (Targher and Byrne, 2015; Valenti and Baselli, 2019). Because of the complicated pathogenesis of MAFLD, agents approved by the Food and Drug Administration for MAFLD are lacking (Friedman et al., 2018). Therefore, exploration of effective and safe novel strategies to treat MAFLD is valuable.

The beneficial effects of TCMs have long been proven to treat chronic metabolic diseases (Liu et al., 2015), such as Ganpi (modern name: MAFLD). The details of the mechanisms and bioactive components of most TCMs have not yet been elucidated. Similarly, the anti-hyperlipidaemic mechanisms of QGS, which has a long history of clinical use to treat this disease, have not been fully investigated. In our study, the extract of QGS was collected and identified to contain mainly polysaccharides, which comprised 75.5% of the total extract composition. Additionally, the ratio of galacturonic acid in the QGS polysaccharide was 18.1%, suggesting that it was an acidic polysaccharide. Additionally, proteins and polyphenols were determined to be present in smaller proportions. Thus, polysaccharides are the main bioactive ingredient. This finding is consistent with other reports that polysaccharides are the main contributors to the lipid-lowering effects of *Polygonatum sibiricum* (National Pharmacopoeia Committee, 2010) and *Morus alba L* (Wen et al., 2019). Furthermore, the monosaccharide composition was measured, and glucose exhibited a molar ratio of 0.896, indicating that it is the main monosaccharide in QGS. In the infrared spectra, peaks at 3,328 and 2,927 cm^−1^ were identified as absorption peaks of polysaccharides, and the modes of linkage among monosaccharides were identified as β-glycosidic bonds and α-glycosidic bonds. A previous study showed that α-/β-glycosidic bonds in polysaccharides are strongly associated with antioxidant activity (Chen and Huang, 2018). Excess oxidant stress is a crucial cause of hepatic damage and results in simple steatosis. Thus, the effect of QGS in the treatment of MAFLD may be attributed to its antioxidant properties (Ferramosca et al., 2017).

In our animal experiments, the high-fat diet provided more energy than the normal diet, and the consumption of food intake was lower in the HFD group than in the NC group and exhibited no difference between the HFD and QGS groups. Additionally, the level of body weight in the HFD group was increased significantly after feeding the high-fat diet but slightly decreased with QGS administration. Thus, QGS could mainly influence the metabolism of TC and TGs against the development of MAFLD. QGS polysaccharides lowered the levels of TC and TGs in both plasma and liver tissue and reduced the LDL-C levels in plasma. Additionally, QGS polysaccharides slightly ameliorated liver injury by decreasing the plasma AST and ALT levels in rats with MAFLD. Overall, these data indicate that QGS possesses beneficial effects in treating hepatic lipid accumulation and liver injury. The results of chemical indexes are enhanced by H&E and oil red O staining results of liver tissue.

To investigate the potential functional mechanism of QGS, obtaining target genes related to the progression and treatment of MAFLD was necessary. RNA sequencing, a new strategy to screen DEGs between sample groups, has been widely performed to identify potential genes involved in the development and treatment of MAFLD (Govaere et al., 2020). In our study, DEGs between the NC and HFD groups, the HFD and QGS groups, and the NC and QGS groups were identified using DESeq2 (Love et al., 2014). Subsequently, a clustering heatmap was created for the DEGs and indicated that QGS reversed the trends in gene expression induced by high-fat diet feeding, causing the expression profile in the QGS group to be similar to that in the NC group. The altered genes associated with different processes should be further analyzed to obtain more information concerning the development of MAFLD.

To determine which biological functions and metabolic pathways involved in MAFLD were affected by QGS treatment, GO and KEGG enrichment analyses were performed. the terms lipid metabolic process (GO: 0019216) and response to nutrient levels (GO: 0031667) were enriched in the biological process category. These processes are tightly involved in lipogenesis. Hepatic lipid metabolism is usually associated with the occurrence and treatment of MAFLD. Disruption of the balance of lipid metabolism in the liver can lead to lipid accumulation and consequently MAFLD (Pei et al., 2020). In our study, excess intake of a high-fat diet disrupted the balance of cholesterol and TG synthesis and led to dyslipidaemia. Additionally, the DEGs were mostly enriched in the non-alcoholic fatty liver disease pathway (hsa04932), which is a crucial signaling pathway for the regulation of MAFLD. In our study, 24 genes regulated by QGS were mapped to the non-alcoholic fatty liver disease pathway. Only the expression of Srebp-1 was downregulated by QGS treatment after high-fat diet-mediated model construction. Srebp-1, a transcription factor, affects the activity and expression of fatty acid synthase (Wang et al., 2020). Additionally, the TNF signaling pathway (hsa04668) was also enriched and affected inflammation in MAFLD. Recent studies suggest that anti-inflammatory agents may be useful for MAFLD treatment (Wang et al., 2021). Thus, QGS may intervene in TNF-α to drive the progression of MAFLD to NASH, and verification for this purpose will be performed in future studies.

Srebps have two isoforms: Srebp-1 and Srebp-2. Srebp-1 preferentially activates the transcription of genes required for fatty acid and triglyceride synthesis. By contrast, Srebp-2 regulates the transcription of genes related to cholesterol synthesis and uptake (DeBose-Boyd and Jin, 2018). Lipid droplets, formed with triglycerides that are digested in the form of fatty acids in the liver, store neutral lipids during times of energy excess, whose prolonged storage leads to MAFLD (Nina L. Gluchowski et al., 2017; Sahini and Borlak, 2014). Srebp-1 acts as a crucial regulator of lipogenesis progression by affecting fatty synthesis genes and adjusting the levels of TGs in the plasma and liver. Thus, in our study, we investigated the activation of Srebp-1 in the liver of MAFLD rats with or without QGS intervention. A high-fat diet was used to promote the expression and activation of Srebp-1 and then the consequent transfer of Srebp-1 to the nucleus from the cytoplasm (Hao et al., 2010; Moon, 2017). When Srebp-1 enters the nucleus, it combines with the promoter region of fatty acid synthesis and related genes associated with lipogenesis, leading to the synthesis of TGs and formation of lipid droplets in the liver (Porstmann et al., 2005). Many studies have proven that inhibition of the expression and activation of Srebp-1 is an efficient method for MAFLD amelioration (Ziamajidi et al., 2013). In our study, immunofluorescence and Western blotting demonstrated that less Srebp-1 was recruited to the nucleus in the QGS group than in the HFD group, indicating that activation of Srebp-1 was inhibited and that the expression of related genes such as fatty-acid synthase and acetyl-coenzyme A carboxylase was suppressed (DeBose-Boyd and Jin, 2018). Thus, QGS contributed to the inhibition of lipogenesis and amelioration of lipid accumulation in the liver. Although Srebp-1 was predicted as an important target gene in MAFLD rats with QGS intervention, limitations persist because of the measurement method, and further biological verification is required. The lipid accumulation-lowering and anti-inflammatory effects may be associated with the expression and activation of Srebp-1. However, the mechanism by which the QGS formula treats MAFLD rats has not been fully explained. Thus, we will examine the biological function of QGS from various perspectives in future studies.

Furthermore, our results were supported by several previous studies. Multiple studies have also demonstrated that polysaccharides from *Polygonatum sibiricum and Morus alba L* exhibit hypolipidaemic activity by inhibiting the progression of cholesterol and TG synthesis (C-G Liu et al., 2017; Yang et al., 2015). Likewise, *Cichorium intybus* polysaccharides inhibit lipogenesis by modulating AMPK (Wu et al., 2018). Some studies have also supported that the expression of Srebp-1 and fatty acid synthase is significantly decreased following treatment with the ethanol extract of *Polygonatum sibiricum* (Ko et al., 2015) and *Cichorium intybus* polysaccharide (Ziamajidi et al., 2013). Notably, ethanol extracts of *Citrus reticulata Blanco* can ameliorate lipid accumulation in the liver (Ke et al., 2020). These findings are consistent with our experimental results, suggesting that QGS polysaccharide ameliorates high-fat diet-induced disorders of lipid metabolic processes in rats. Furthermore, previous studies have shown that *Glycyrrhiza uralensis Fisch* extract mitigates TNF-α overproduction in rats with MAFLD (Jung et al., 2016; Gou et al., 2020). *Cirsium setosum* extract exerts obvious anti-inflammatory effects, and this herb is also often utilized to prevent cardiovascular disease (Wang et al., 2019). Thus, QGS may protect against inflammation resulting from lipid accumulation, a finding that must be comprehensively investigated in further studies.

Recent studies have demonstrated that CVD is the major cause of death in MAFLD patients (Athyros et al., 2017). For MAFLD/NASH patients with a high CVD risk, statins, as first-line drugs for lowering the lipid content, have been widely utilized. However, treatment resistance and intolerance are the main limitations of statin use with long-term administration (Doumas et al., 2019). Importantly, statins have no effects on hepatic fat accumulation. Herein, the QGS formula comprising six herbs obviously mitigated excess lipid accumulation in the liver. In a subsequent study, the combination of QGS and statins probably became an adjunct therapeutic strategy for MAFLD patients with CVD risk and expanded the application of QGS in clinical practice.

The presence of MAFLD in T2DM patients is strongly correlated with more severe dyslipidaemia and insulin resistance in hepatic and adipose tissue than in T2DM patients without MAFLD (Tilg et al., 2017). An explanation for the association between liver lipid accumulation and insulin resistance is that liver fat accumulation is directly generated from excess lipid availability and leads to insulin resistance (Roden, 2006). However, first-line blood glucose-lowering drugs, such as metformin, obviously decrease both hepatic and peripheral insulin resistance but have no effects on reducing liver fat accumulation (Tilg et al., 2017). The QGS formula significantly ameliorates liver fat accumulation by inhibiting the expression and activation of Srebp-1 and might alleviate insulin resistance by reducing fat accumulation. The QGS formula, as a supplemental therapeutic strategy to blood glucose-lowering drugs such as metformin, may be a novel lipid-reducing therapeutic strategy to treat T2DM patients with MAFLD.

## Conclusion

Our data suggest that QGS effectively lowers lipids in both serum and liver and partly protects liver function in MAFLD rats. The lipid-lowering effects are mediated by decreased TC and TG production by regulating the expression and activation of Srebp-1.

## Data Availability

The data presented in the study are deposited in the NCBI repository, accession number (PRJNA726732).

## Abbreviations

ALT, plasma alanine aminotransferase; AST, aspartate aminotransferase; CVD, cardiovascular disease; DEGs, differentially expressed genes; GO, Gene Ontology; H&E, hematoxylin and eosin; HDL-C, high-density lipoprotein cholester; HFD, high-fat diet; KEGG, Kyoto Encyclopedia of Genes and Genomes; LDL-C, low-density lipoprotein cholesterol; MAFLD, metabolism-associated fatty liver disease; NC, normal control; NASH, non-alcoholic steatohepatitis; QGS, Qing Gan San; Srebp-1, sterol regulatory element-binding protein-1; T2DM, Type 2, diabetes mellitus; TC, total cholesterol; TCMs, traditional Chinese medicines; TG, triglycerides; TNF-α, tumor necrosis factor-α.
