# Supplementary figures and images for: Prediction of Srebp-1 as a Key Target of Qing Gan San Against MAFLD in Rats via RNA-Sequencing Profile Analysis
Source: Front Pharmacol. 2021 Jul 5;12:680081. doi: 10.3389/fphar.2021.680081 (PMC8289482; doi:10.3389/fphar.2021.680081)

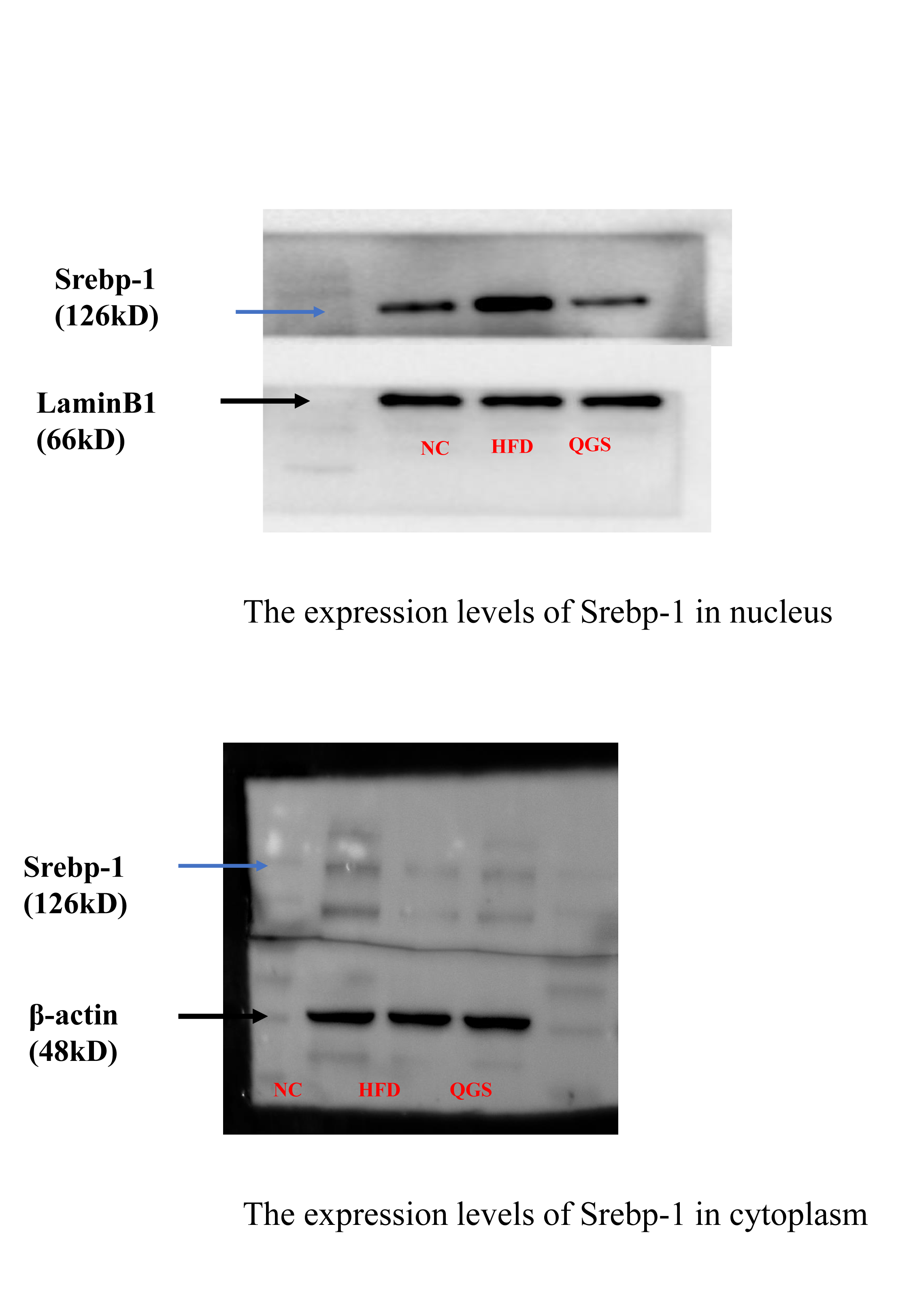

Supplement: Supplementary file 1 [file Image1.TIF]
